# 气流辅助熔融态聚合法制备荧光碳点检测医疗废水中四环素

**DOI:** 10.3724/SP.J.1123.2024.03001

**Published:** 2024-11-08

**Authors:** Ziwei LIU, Lanxiu NI, Jie CHI, Yingxi QIN, Zhenjia SHI, Yu WANG, Huangzhao WEI, Liang FENG, Chenglin SUN

**Affiliations:** 1.中国科学院大连化学物理研究所, 辽宁 大连 116023; 1. Dalian Institute of Chemical Physics, Chinese Academy of Sciences, Dalian 116023, China; 2.中国科学院大学, 北京 100049; 2. University of Chinese Academy of Sciences, Beijing 100049, China; 3.东北大学, 辽宁 沈阳 110819; 3. Northeastern University, Shenyang 110819, China; 4.桂林理工大学, 广西 桂林 541004; 4. Guilin University of Technology, Guilin 541004, China; 5.博识(大连)信息技术有限公司, 辽宁 大连 116023; 5. Boshi (Dalian) Information Technology Co., Ltd., Dalian 116023, China

**Keywords:** 碳点, 荧光法, 四环素, 医疗废水, carbon dots (CDs), fluorescence, tetracycline, medical wastewater

## Abstract

对环境水样中四环素(TC)污染物的控制措施和环保管理均需要精确获得环境介质中TC污染物的浓度。碳点(CDs)是一种新兴的荧光材料,它具有易制备、低成本、低毒性及良好的生物相容性等优点,在TC检测领域广受关注。本文以丙三酸(tricarboxylic acid)与乙二胺(ethylenediamine)为前驱物,采用气流辅助熔融态聚合法合成了具有蓝色荧光的TE-CDs,并建立了基于TE-CDs的TC检测方法。对所制备的TE-CDs形貌及结构进行表征,透射电镜结果表明TE-CDs分散较好,平均直径为(2.43±0.48) nm。X射线衍射结果显示TE-CDs具有无定形碳结构。红外光谱及X射线光电子能谱结果表明TE-CDs表面富含氨基、羟基和羧基等亲水基团,说明TE-CDs具有良好的水溶性,有利于实现对医疗废水中TC的检测。TE-CDs具有激发依赖性,最大激发波长和发射波长分别为368 nm和448 nm。TC可有效地将TE-CDs的蓝色荧光淬灭,利用此性质可实现对TC的检测。另外,TE-CDs对TC响应灵敏,在较宽的pH范围内荧光强度稳定。在TC质量浓度从2 mg/L增加到200 mg/L时, TE-CDs的荧光强度呈现不同程度的降低,直至完全淬灭,淬灭机制为动态淬灭。在4~20 mg/L范围内,TC质量浓度与TE-CDs荧光淬灭程度呈现良好的线性关系(*R*^2^=0.9978),检出限为0.2 mg/L。所制备TE-CDs响应灵敏,荧光稳定性较优,选择性较好,采用该方法对医疗废水中的TC定量检测,回收率为96.5%~119.8%,相对标准偏差(RSD)为0.8%~2.6%,该方法省时简易,重复性好,具有良好的实际应用价值。

## Abstract (en)

Tetracycline (TC) is one of the most important therapeutic drugs that is widely used in hospitals. However, its harmful effects on human health and various ecosystems cannot be ignored. Owing to its poor metabolic activity and low biodegradability, TC commonly discharges as the parent compound and accumulates readily in sludges and soils by precipitation from wastewater, which can induce the evolution of antibiotic-resistant bacteria; therefore, it has been listed as one of the new pollutants with potential ecotoxicological risk. The control measures and environmental management of TC pollutants in environmental water samples require precise determination of TC pollutant concentrations. Carbon dots (CDs) are an emerging type of fluorescent material with numerous advantages such as easy preparation, low cost, low toxicity, and good biocompatibility. Consequently, they have attracted widespread attention in the field of TC detection. Herein, we synthesized TE-CDs with good blue-fluorescence performance via flow-assisted melt polymerization using tricarboxylic acid and ethylenediamine as raw precursors. The morphology and structure of the prepared TE-CDs were characterized. The transmission electron microscopy (TEM) results showed that the prepared TE-CDs were well dispersed, with an average diameter of (2.43±0.48) nm. The X-ray diffraction (XRD) results showed that the TE-CDs had an amorphous carbon structure. Infrared spectroscopy and X-ray photoelectron spectroscopy (XPS) characterizations showed that the surface of the TE-CDs was rich in hydrophilic groups, such as amino, hydroxyl, and carboxyl groups, which indicated that TE-CDs had good water solubility and were advantageous for detecting TC in medical wastewater. Subsequently, the optical properties of the TE-CDs were investigated. The fluorescence emission spectra of the TE-CDs were recorded at various excitation wavelengths. The emission spectra of the TE-CDs exhibited excitation wavelength dependence and when the excitation wavelength changes from 300 nm to 400 nm, their fluorescence intensity decreased to varying degrees. The TE-CDs exhibited optimal fluorescence intensity at an excitation wavelength of 368 nm, while the emission wavelength was 448 nm.

TC could effectively quench the blue fluorescence of the CDs, and by utilizing this property, the detection of TC concentration could be achieved. After the addition of TC, the fluorescence of the system immediately reached an extreme value, and no significant change was observed within 10 min. An incubation time of 20 s was selected to obtain precise results. Additionally, the TE-CDs exhibited stable fluorescence intensity over a wide pH range. The fluorescence stability of the TE-CDs was investigated, and no significant change in fluorescence intensity was observed after standing for 10 d, indicating that the prepared TE-CDs had excellent fluorescence stability. The fluorescence intensity of the TE-CDs decreased to varying degrees within the range of 2-200 mg/L TC until complete quenching occurred. TC mass concentration in the range of 4-20 mg/L showed a good linear relationship (*R*^2^=0.9978) with the fluorescence quenching intensity of the TE-CDs. The limit of detection was 0.2 mg/L. A preliminary investigation was undertaken to explore the quenching mechanism of the TE-CDs fluorescence by TC. Upon addition of TC, a significant reduction in the fluorescence lifetime of the TE-CDs was observed. During the quenching process, no new substances were observed by UV absorption spectroscopy. Additionally, no significant changes in the ^1^H NMR spectra of the TE-CDs were noted before and after the addition of TC, indicating the absence of an interaction between the TE-CDs and TC. Therefore, the quenching mechanism may involve dynamic quenching. The selectivity and anti-interference ability of the developed method were evaluated; in the presence of interfering substances, TC quenched the fluorescence of the TE-CDs, indicating that the TE-CDs had good selectivity and anti-interference performance towards TC. The method was applied to the quantitative detection of TC in medical wastewater, with recoveries of 96.5%-119.8% and relative standard deviations of 0.8%-2.6%. In conclusion, the analytical performance of the proposed method is comparable with that of previously reported detection methods; moreover, the method has the advantages of low operational cost, simple preparation process, time-saving, and good repeatability. Therefore, the TE-CDs can be used as chemical sensors for the detection of TC in medical wastewater and have good practical applications.

四环素(tetracycline, TC)作为一种广谱抗生素药物,常用于治疗人类和动物的革兰氏阳性菌、革兰氏阴性菌感染^[1,2]^,是目前使用最广泛的治疗药物之一^[3]^。但是,TC进入人体后,仅有少部分能被机体代谢或吸收,高达75%的TC以母体化合物形式通过代谢排出体外,释放到自然环境中,经水循环最终在各类水体或水生生物中积累、富集^[4]^。当人类饮用或食用含有TC的水或食物时,会产生胃肠道紊乱、肝脏损伤、牙齿发育不良等危害^[5,6]^。此外,自然界中TC的残留还会引起耐药菌产生等诸多问题。抗生素是医院使用的最重要的治疗药物之一。当前,由于缺乏TC等相关抗生素的排放标准,经过污水处理系统排放的废水中仍存在抗生素残留,因此,为了防止TC过量进入自然界,对医疗废水中TC的浓度变化进行密切监测十分关键^[7]^。目前,已建立了多种检测TC的方法,如高效液相色谱法^[8]^、高效液相色谱-质谱法^[9,10]^、电化学法^[11]^、酶联免疫吸附法^[12]^、表面增强拉曼光谱法^[13]^等。虽然这些方法具有较高的灵敏度和准确性,但由于检测成本高、周期长、操作复杂等问题导致难以建立广泛的TC监测网络。为了解决这一问题,首要任务是开发一种高效、快速且简便的TC检测方法。

荧光测定法(fluorometry)作为一种新型检测方法,在过去20年中引起了广泛关注。由于荧光测定法具有灵敏度高、预处理简单、成本低等优点,在分析检测领域得到了快速发展^[14]^。在众多荧光传感材料中,碳点(carbon dots, CDs)是一种新兴的纳米荧光材料,它具有易制备、低成本、低毒性、高抗光漂白性、良好的生物相容性和可分散性等优点,因此,在基于荧光测定法的抗生素检测中崭露头角。到目前为止,基于CDs的抗生素荧光传感器多有报道^[5,15⇓⇓⇓-19]^,例如Wang等^[5]^以葡萄糖和乙二胺为原料通过水热法制备高发光氮掺杂CDs,用以检测3种四环素类抗生素,检出限为0.117~0.344 μmol/L;将此方法应用于血清和牛奶样品中3种四环素类抗生素的检测,回收率为96.5%~119.8%。再如Yan等^[18]^在室温下以乙二醛为碳源,邻苯二胺为碳氮源简便合成了发黄绿色荧光的CDs,该方法已成功用于测定四环素片中TC的含量。此外,Nie等^[19]^合成了荧光CDs并将其与铕离子(Eu^3+^)螯合,实现了对TC的定量分析,检出限低至16.4 nmol/L,将该CDs用于实际环境水样中TC的分析,其回收率为95.7%~109.8%。然而,大多数报道中CDs制备过程相对复杂,产率低,不利于CDs作为荧光传感器在实际工作中的大范围应用。

本文以丙三酸(tricarboxylic acid)与乙二胺(ethylenediamine)为前驱物,采用一步气流辅助熔融态聚合法合成了具有蓝色荧光的TE-CDs。通过分散与反相沉淀,便可实现TE-CDs与杂质的快速分离与纯化。制备过程操作简单,周期短,所制备的TE-CDs具有良好的水溶性与荧光稳定性。因TE-CDs对TC响应灵敏,将此方法用于检测医疗废水中TC的残留, 结果表明该方法具有实用性,在辅助构建大范围TC监测网络领域具有较大潜力。

## 1 实验部分

### 1.1 仪器与试剂

SRJK-2-13高温燃烧管式炉(天津市泰斯特仪器有限公司); ME104电子天平(梅特勒-托利多仪器(上海)有限公司); DZF-6020真空干燥箱(昆山一恒仪器有限公司); SK250H超声波清洗器(上海科导超声仪器有限公司); F-4600荧光分光光度计(日本日立高新技术公司); TU-1901双光束紫外-可见分光光度计(北京普析通用仪器有限责任公司); FEI Tecnai F20透射电子显微镜(美国FEI公司); INVENIO S红外光谱仪(德国布鲁克公司); X射线衍射仪(荷兰帕纳科公司); X射线光电子能谱仪(美国Thermo Scientific K-Alpha公司)。

丙三酸、四环素、磺胺噻唑(STZ)、磺胺多辛(SD)、磺胺甲噁唑(SMZ)、利奈唑胺(LZ)、氯霉素(CPL)(上海麦克林生化科技股份有限公司),乙二胺(国药集团化学试剂有限公司),甲醇、二氯甲烷(广东光华科技股份有限公司),碳酸氢钠、硫酸钠、氯化铵、氯化钙、氯化钾、葡萄糖(天津市科密欧化学试剂有限公司),乙二胺四乙酸(EDTA)、氯化钠、氯化锌(上海阿拉丁生化科技股份有限公司),氯化镁(上海迈瑞尔生化科技有限公司),氯化铝、氯化铜、甘氨酸、组氨酸(阿法埃莎(中国)化学有限公司),所有试剂均为分析纯,使用前不进行任何处理。

### 1.2 TE-CDs制备

TE-CDs采用气流辅助熔融态聚合法合成。首先,称取1.76 g丙三酸,加入0.72 g乙二胺,搅拌研磨混合,将混合物转移至石英舟中,随后放入高温管式炉,以100 cm^3^/min的流速通入高纯N_2_,保持15 min后开始升温。在230 ℃下煅烧2 h,冷却后得到固体块状的产物。将所得固体块状产物研磨成粉末,依次加入5 mL甲醇并搅拌30 min、加入5 mL二氯甲烷并搅拌30 min,过滤,使用5 mL二氯甲烷将沉淀物洗涤3次。最后在60 ℃下真空干燥6 h,即可得到纯化后的TE-CDs固体粉末(约1.73 g),产率约为70%。

### 1.3 TE-CDs检测TC

移取1 mL TE-CDs溶液(1 mg/mL)至比色皿中,分别添加质量浓度为0~200 mg/L的TC溶液1 mL, 反应20 s后,测其发射光谱(激发波长为368 nm),荧光强度记作*F*,不添加TC时记为*F*_0_,平行测定3次取平均值,依据*F/F*_0_与TC浓度的线性关系绘制标准曲线。

### 1.4 样品的收集与处理

医疗废水取自大连医科大学附属第二医院,收集后,采用定性滤纸过滤3 L废水样品以去除颗粒物,并加入15 g NaCl,利用钌钛电极在15 V的恒定电势下进行电催化氧化。取电解后的1 L废水,加入2.0202 g Na_2_SO_3_中和余氯。处理后的水样均在室温下储存。考虑到医疗废水中存在的金属离子会与目标抗生素螯合^[20,21]^,在水样中加入0.5 g/L EDTA释放目标抗生素。

## 2 结果与讨论

### 2.1 TE-CDs的制备及表征

目前,CDs的合成方法主要为自下而上的水热/溶剂热法等,如Jia等^[22]^采用一锅水热法制备N-CDs的过程中,首先在高压反应釜中进行反应,随后经过过滤、透析、冷冻干燥处理最终得到N-CDs固体粉末,制备过程繁琐耗时。本研究所制备的TE-CDs采用气流辅助熔融态聚合法,即在持续通入N_2_的环境中对前驱物进行熔融态聚合,即可获得TE-CDs粗产物。经过简单的共分散与沉淀,即可实现TE-CDs与杂质的快速分离纯化。TE-CDs的产率达到了70%。整个制备过程操作简单、周期短、能耗低、产率高,有利于使用TE-CDs荧光传感器建立大范围TC监测网络。

TE-CDs的透射电镜(TEM)如图1a所示,分散性较好,无明显晶格条纹,尺寸分布范围为1.6~3.2 nm,平均直径为(2.43±0.48) nm(图1b)。TE-CDs的X射线衍射(XRD)如图2a所示,在2*θ*=19.7°左右出现一个宽的衍射峰,表明所制备的TE-CDs具有无定形碳的结构^[23]^。

**图1:**
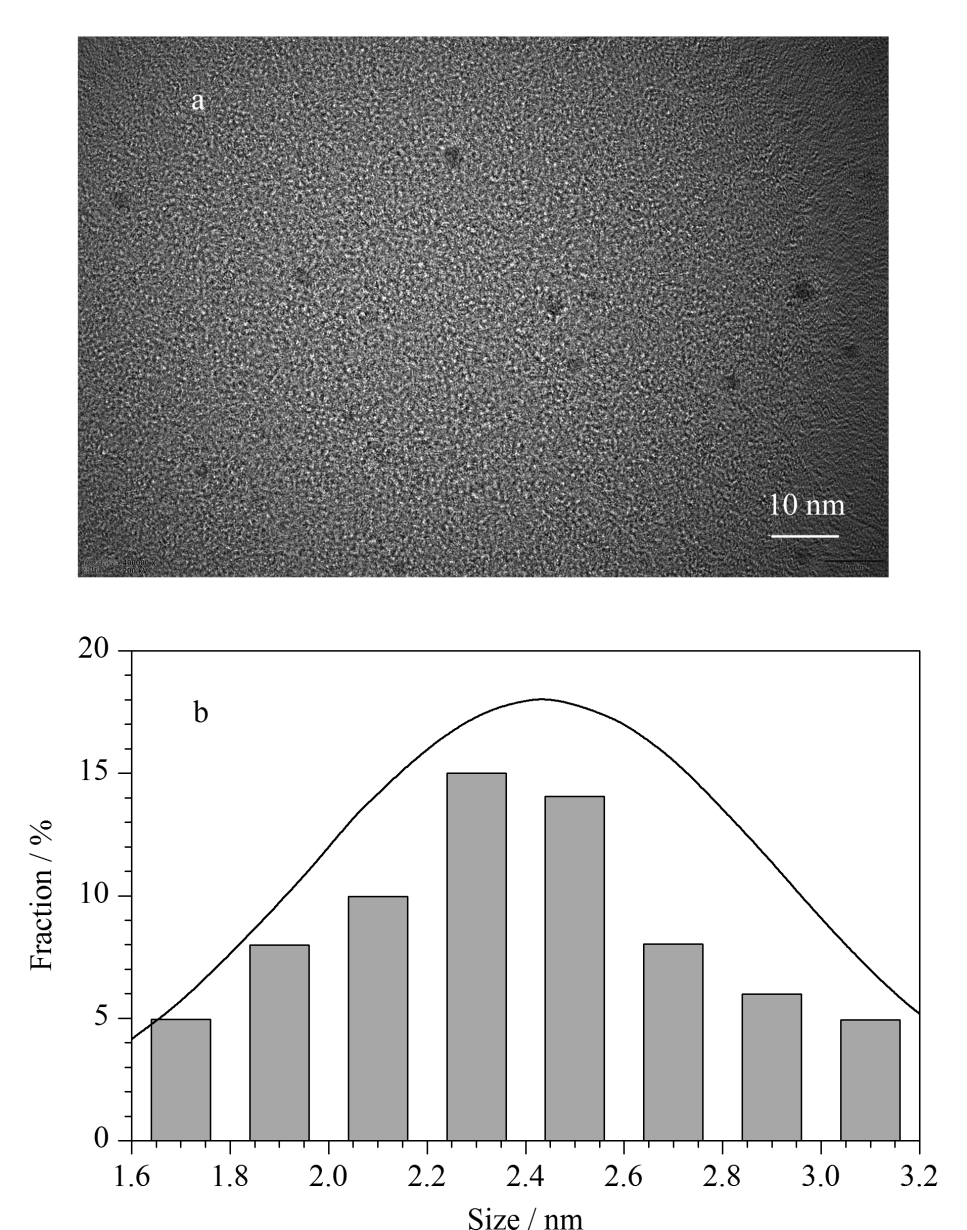
TE-CDs的(a)TEM图和(b)直径分布

**图2:**
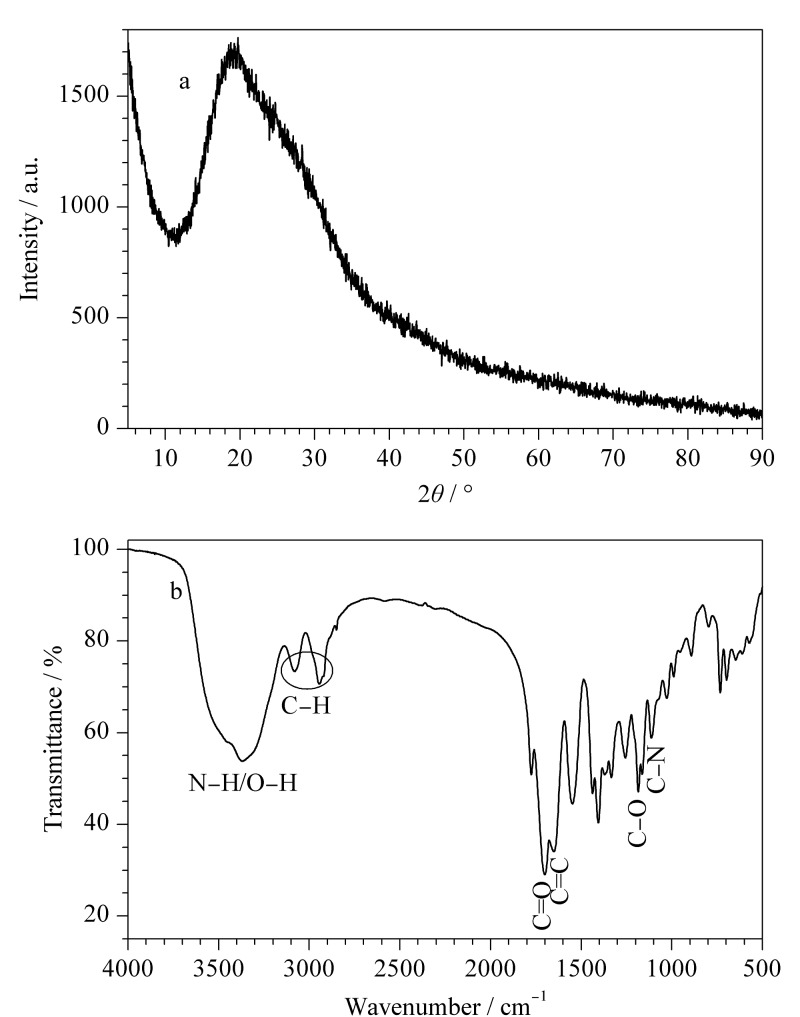
TE-CDs的(a) XRD图和(b)红外光谱图

红外光谱(IR)显示(图2b), TE-CDs在3368 cm^-1^处呈现O-H和N-H伸缩振动峰,2934 cm^-1^、3073 cm^-1^处的峰值归因于C-H的振动,1705 cm^-1^处的强峰归属于C=O键的伸缩振动,1676 cm^-1^处的吸收峰是由C=C键的伸缩振动引起,1182 cm^-1^、1105 cm^-1^处分别为C-O、C-N的伸缩振动峰^[24]^。

为了进一步探究TE-CDs的化学构成和表面化学性质,对TE-CDs进行X射线光电子能谱(XPS)表征。如图3a所示,TE-CDs全谱图中,C 1*s*、N 1*s*和O 1*s*含量分别为67.96%、12.58%、19.46%。对XPS全谱图进行分峰,TE-CDs的C 1*s*分峰拟合图如图3b所示,键能分别为284.80、285.79和288.04 eV,分别对应C-C/C=C、C-N/C-O及C=O/C=N。N 1*s*的分峰拟合图如图3c所示,键能分别为399.49、400.22 eV,分别对应C-N、N-H。O 1*s*的分峰拟合图如图3d所示,键能分别为531.19、532.51 eV,分别对应C-O、C=O^[25⇓-27]^。

**图3:**
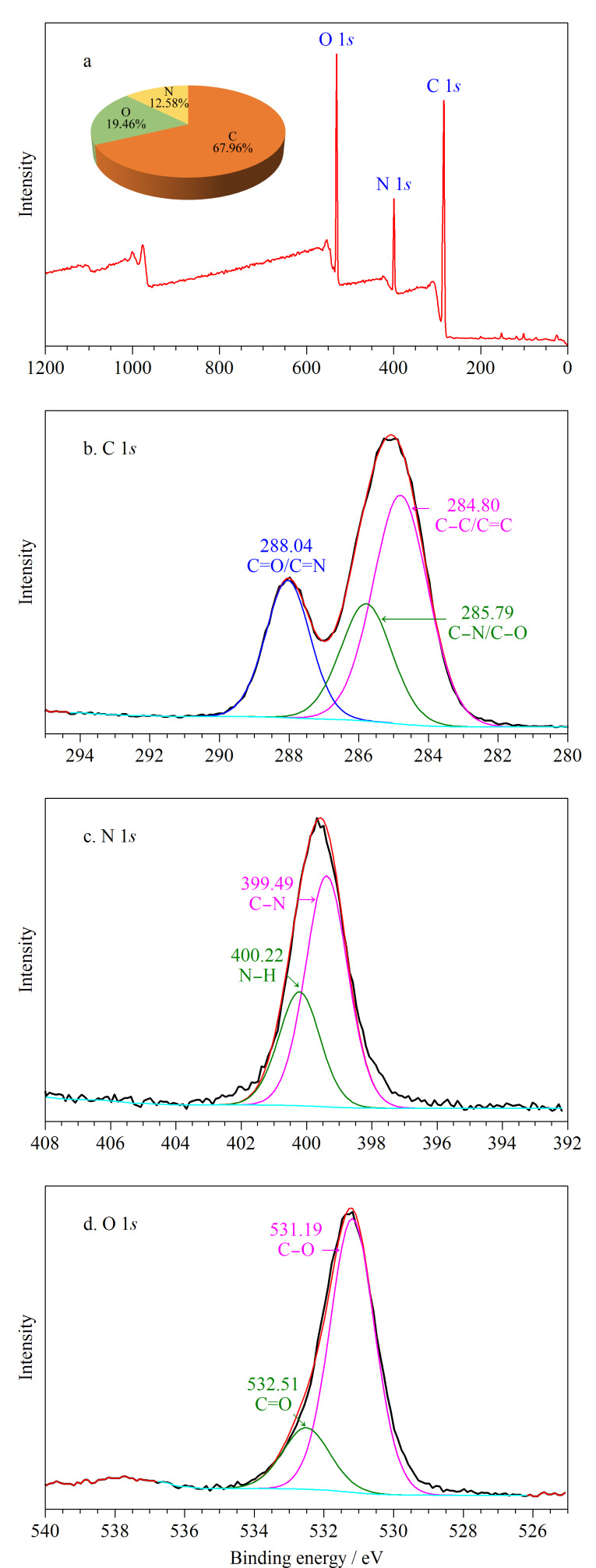
TE-CDs的(a)XPS全谱图和(b)C 1*s*、(c)N 1*s*、(d)O 1*s*峰拟合图

IR与XPS结果表明,TE-CDs表面富含氨基、羟基和羧基等亲水基团,说明TE-CDs具有良好的水溶性,有利于实现对医疗废水中TC的检测。

### 2.2 TE-CDs的光学性质

为探究所制备TE-CDs的光学性质,分别测定了TE-CDs的紫外可见吸收光谱和荧光光谱。如图4a插图所示,TE-CDs溶液呈无色透明态,在365 nm紫外灯照射下呈现明亮的蓝色荧光。如图4a所示,质量浓度为500 mg/L的TE-CDs在紫外吸收区呈现出明显吸收峰,可归因于TE-CDs内部的π-π^*^跃迁^[5,13,28,29]^。当激发波长由300 nm增加至400 nm时,与大多数已报道文献类似^[1,29,30]^, TE-CDs发射光谱表现出激发波长依赖性。发射峰出现蓝移和红移现象且荧光强度呈现不同程度的降低,在激发波长为368 nm处荧光强度最大,发射波长为448 nm,如图4b所示,可能是由于表面缺陷及纳米颗粒尺寸变化所致^[31,32]^。因此本研究选取激发波长为368 nm、发射波长为448 nm进行后续实验。此外,考察了存储时间对TE-CDs荧光稳定性的影响,如图4c所示,在室温条件下储存10天内,TE-CDs荧光强度无明显变化,显示出较优的稳定性。

**图4:**
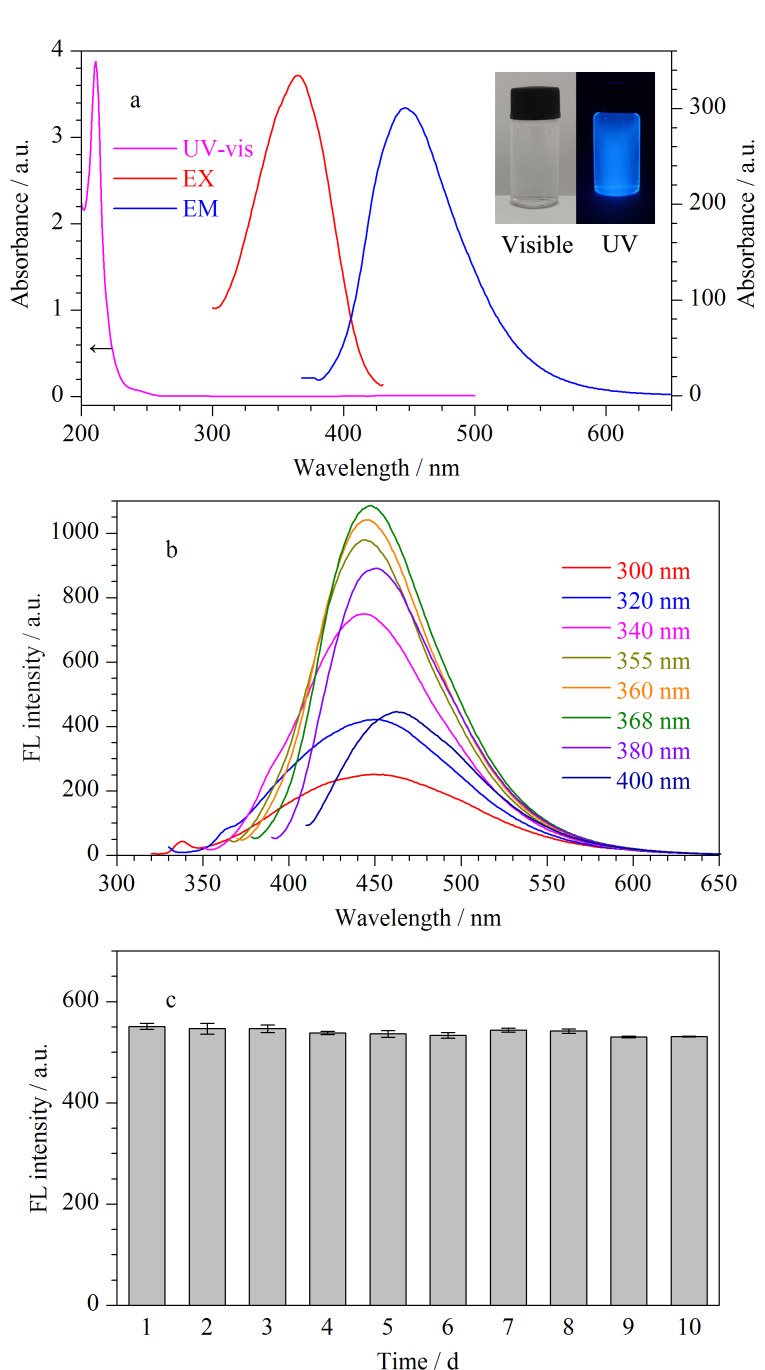
(a)TE-CDs的紫外-可见吸收光谱、荧光激发和发射光谱 (插图:可见光和紫外灯照射), (b)不同激发波长下TE-CDs的荧光强度,(c)TE-CDs的荧光稳定性(*n*=3)

### 2.3 TC的荧光响应

考察了响应时间与pH对TE-CDs荧光响应的影响。首先测定了TE-CDs对TC的响应时间,如图5a所示。在添加5、20、50、70 mg/L 4个水平的TC后,TE-CDs (500 mg/L)的荧光立即表现出明显淬灭,并在之后10 min内荧光强度保持稳定。为了保证检测体系的稳定性,选用20 s作为响应时间。随后考察了pH对TE-CDs荧光强度的影响,如图5b所示,在pH 3~11范围内,TE-CDs荧光强度变化较小,因此,选用pH=7进行实验。如图5c所示,在TE-CDs水溶液(500 mg/L)中加入不同质量浓度的TC(2~200 mg/L), TE-CDs荧光强度逐渐下降至完全淬灭。图5d考察了*F/F*_0_比值与TC质量浓度的线性关系,在4~20 mg/L的TC质量浓度范围内,*F/F*_0_比值与TC质量浓度([TC])呈良好线性,如图5d插图所示,两者的线性方程为*F/F*_0_=-0.0199[TC]+0.9565,相关系数(*R*^2^)=0.9978。同时,基于3*σ*/*S*(*σ*是空白样品的标准偏差,*S*是工作曲线的斜率)计算,TE-CDs作为荧光传感器时TC的检出限为0.2 mg/L。

**图5:**
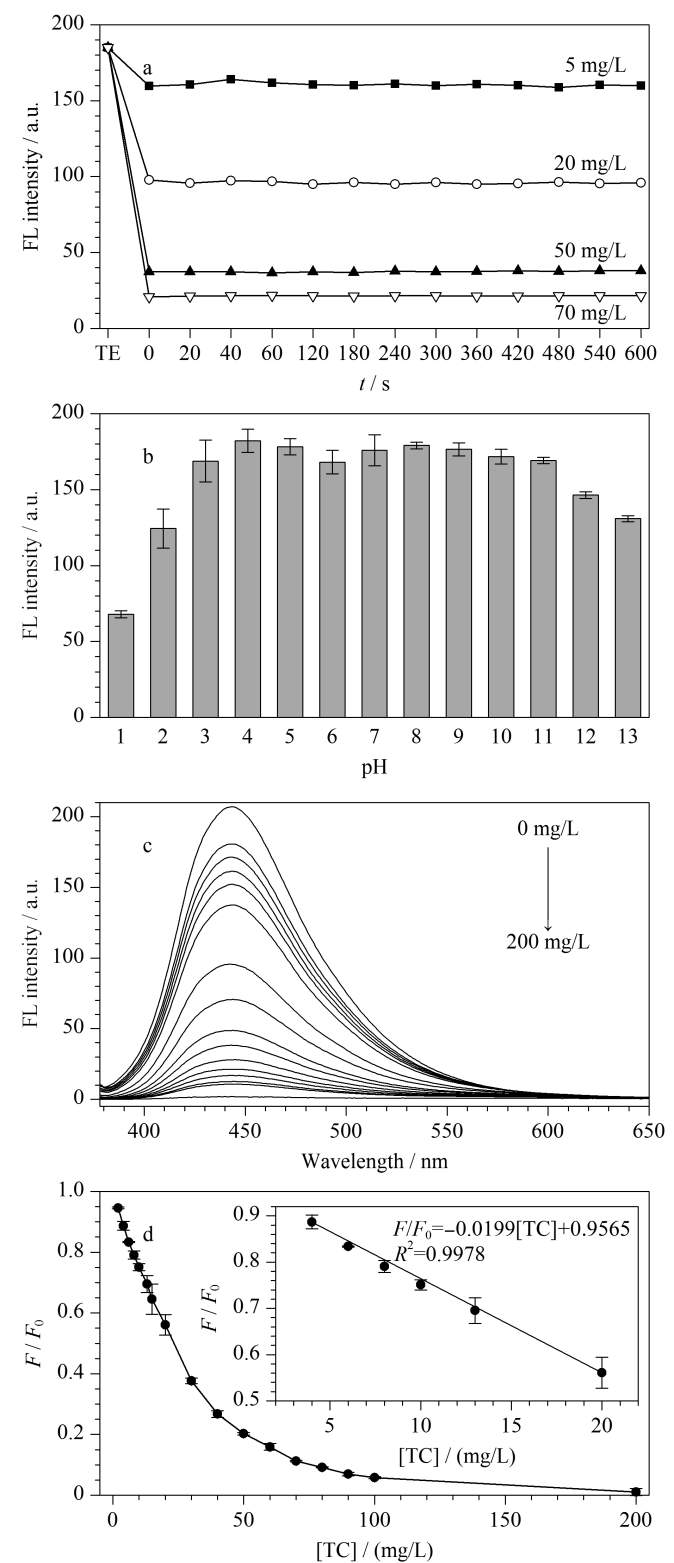
(a)TE-CDs孵化时间及(b)pH对TE-CDs荧光强度 (*n*=3)的影响,(c)TC与TE-CDs淬灭关系图,(d)相对荧光强度与TC质量浓度线性关系(*n*=3)

为了测试TE-CDs是否可以用于医疗废水中TC的测定,选取实际水样中可能存在的潜在干扰物,包括SD、SMZ、STZ、LZ、CPL以及医疗废水中可能共存的 ${{HCO}_{3}}^{-}$ 、 ${{SO}_{4}}^{2-}$ 、 $N{H_{4}}^{+}$ 、Ca^2+^、K^+^、Al^3+^、Mg^2+^、Cu^2+^、Zn^2+^、Na^+^、葡萄糖、组氨酸、甘氨酸。如图6所示,在TE-CDs的水溶液中加入100 mg/L的干扰物质,TE-CDs荧光强度没有发生明显变化。但在干扰物与TC共存的条件下,TE-CDs的荧光明显淬灭。这一结果表明,TE-CDs作为TC检测的荧光传感器具有良好的选择性和抗干扰性。

**图6:**
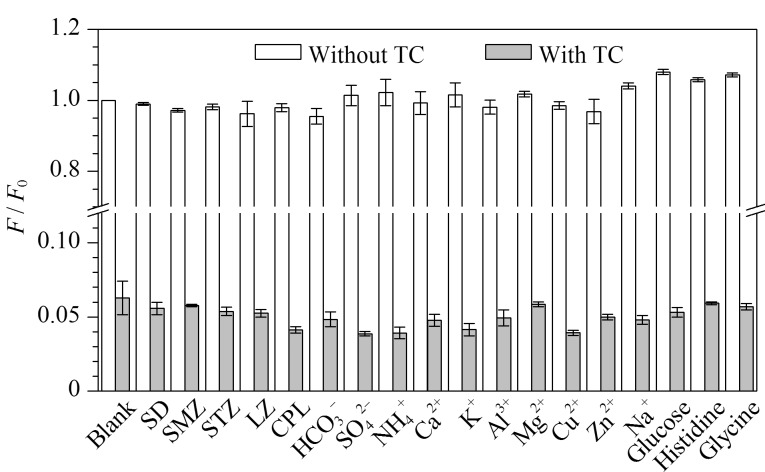
不同干扰物质对TC淬灭TE-CDs的影响(*n*=3)

另外,如表1所示,本方法的分析性能与已报道的检测方法相当,且具有制备材料成本低、制备过程简便、省时等优点,表现出应用于检测实际环境水样中TC含量的潜力。

**表1:** 采用不同碳点的荧光分析法对TC检测效果的对比

CDs	Preparation method and time	Linear range/ (mg/L)		LOD/ (mg/L)	Ref.
N-CQDs	hydrothermal, 52 h	0	-44	0.2	[5]
N-CQDs	hydrothermal, 6 h	0.2	-222	0.07	[6]
Cu-CDs	solvothermal, 27 h	0.9	-14	0.08	[33]
N,S-CDs	hydrothermal, 8 h	0.04	-28	0.02	[34]
TE-CDs	flow-assisted melt	4	-20	0.2	this
	polymerization, 2 h				work

N-CQDs: nitrogen-doped carbon quantum dots; Cu-CDs: copper-doped carbon quantum dots; N,S-CDs: nitrogen and sulfur co-doped carbon dots.

### 2.4 TE-CDs对TC响应的机制

为了明确TE-CDs对TC的响应机制,对加入TC前后TE-CDs的紫外可见吸收光谱、荧光寿命及液体核磁共振变化情况进行分析。如图7a所示,TE-CDs与TC吸收峰位置没有发生变化,仅为吸光度值叠加,表明并无新物质生成。此外,通过测量加入TC前后TE-CDs的荧光寿命,如图7b所示,TE-CDs及TE-CDs+TC的荧光寿命分别为12.60 ns和11.11 ns,表明荧光寿命发生了衰减,推测淬灭机制可能为动态淬灭。为了进一步证明这一推论,利用^1^H NMR分析了TC加入前后TE-CDs中质子峰的变化情况。如图7c所示,TE-CDs中的质子信号并没有因为TC的存在而发生移动,表明TE-CDs与TC没有发生相互作用,证明TC对TE-CDs的荧光淬灭机制为动态淬灭^[6,35,36]^。

**图7:**
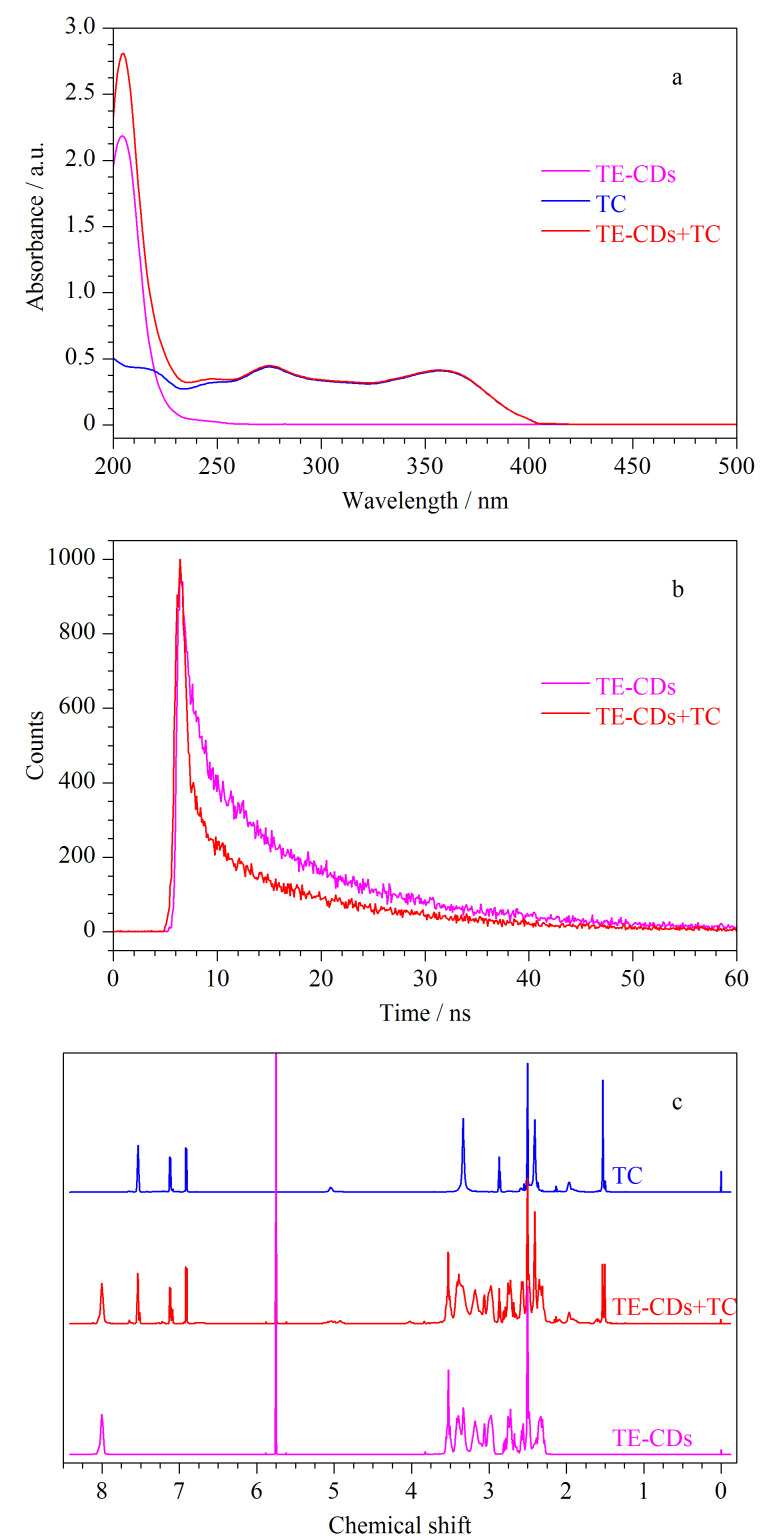
(a)加入TC前后TE-CDs的紫外吸收光谱、(b)荧光寿命与(c)^1^H NMR对比图

### 2.5 实际医疗废水中TC的检测

为了评估所建立方法的可行性,对医疗废水中的TC进行检测,结果表明在实际医疗废水中并未检测到TC。向水样中分别加入5、10、15 mg/L的TC进行加标回收测试。检测结果如表2所示,使用基于TE-CDs的荧光测定法得到的TC加标回收率为96.5%~119.8%,相对标准偏差(RSD)为0.8%~2.6%。结果表明,TE-CDs可以作为荧光传感器用于医疗废水中TC的检测,具有良好的实际应用价值。

**表2:** TC在医疗废水中的加标回收率和相对标准偏差(*n*=3)

Spiked/ (mg/L)	Found/ (mg/L)	Recovery/%	RSD/%
0	0	-	-
5	4.8	96.5	1.1
10	11.3	113.2	2.6
15	18.0	119.8	0.8

## 3 结论

本文建立了基于TE-CDs的TC检测方法。以丙三酸与乙二胺为前驱物,采用气流辅助熔融态聚合法高效合成了具有蓝色荧光的TE-CDs。通过实验证明了TE-CDs作为荧光传感器对TC具有良好的选择性和传感检测能力。将该方法应用于实际医疗废水中TC的检测,结果表明所建立的TC检测方法具有简便、快速及成本低等优点,可为开发大范围TC监测体系提供新思路。
